# Isolation of Porcine Epidemic Diarrhea Virus during Outbreaks in South Korea, 2013–2014

**DOI:** 10.3201/eid2112.150437

**Published:** 2015-12

**Authors:** Hee-Chun Chung, Van Giap Nguyen, Hyoung-Joon Moon, Jee-Hoon Lee, Seong-Jun Park, Ga-Eun Lee, Hye-Kwon Kim, You-Shun Noh, Chan-Hee Lee, Dane Goede, Bong Kyun Park

**Affiliations:** Seoul National University, Seoul, South Korea (H.-C. Chung, J.-H. Lee, G.-E. Lee, Y.-S. Noh, C.-H. Lee, B.K. Park);; Vietnam National University of Agriculture, Hanoi, Vietnam (V.G. Nguyen);; Green Cross Veterinary Products, Yongin, South Korea (H.-J. Moon);; National Forensic Service, Chilgok, South Korea (S.-J. Park);; Institute for Basic Science, Daejeon, South Korea (H.-K. Kim);; Korea Research Institute of Bioscience and Biotechnology, Daejeon, Republic of Korea (H.-K. Kim);; University of Minnesota, St. Paul, Minnesota, USA (D. Goede)

**Keywords:** porcine epidemic diarrhea, PED, porcine epidemic diarrhea virus, PEDV, South Korea, pigs, isolation, viruses, North American strain–like strains, outbreaks, Nidovirales, Coronaviridae, Alphacoronavirus

**To the Editor:** Porcine epidemic diarrhea (PED) is an acute infectious diarrhea caused by the PED virus (PEDV), which belongs to the order *Nidovirales*, family *Coronaviridae*, genus *Alphacoronavirus* (*1*). The virus is transmitted mainly through fecal–oral routes and infects all age groups of pigs; the most severe form of disease occurs in suckling piglets (*1*). PEDV was first reported in South Korea in 1992 (*2*), with the occurrence of an outbreak, and has since circulated with considerable genetic diversity (*1*,^3^). During 2013, PED outbreaks reoccurred in South Korea; however, the emerging PEDVs in these outbreaks were not variants of previous Korean isolates or attenuated vaccine strains (*4*,*5*). We report on a field isolate of a novel emerging PEDV and the isolate’s genetic relationship with other PEDV strains.

During October 2013–June 2014, dead piglets and fecal swabs from 9 provinces of South Korea were sent to the Department of Veterinary Medicine Virology Laboratory at Seoul National University to confirm diagnoses of enteric viral diseases. All samples (30 intestine samples of dead piglets and 16 fecal swabs) were found to be PEDV positive. Attempts to isolate the field strains of PEDV on Vero cell lines followed a previously described protocol with modifications (*6*). An overnight monolayer of Vero cells (80%–100% confluence) was washed twice with 1× phosphate-buffered saline before homogenized samples (0.02 µm filtered) were inoculated with 10% suspension. After 30 min absorption at 37°C with 5% CO_2_, maintenance medium (Dulbecco’s Modified Eagle Medium supplemented with trypsin [10 µg/mL]), yeast extract (0.04%), tryptose phosphate broth (0.6%), and Antibiotic-Antimycotic 100× (4 µl/mL; Gibco, Thermo Fisher Scientific, Grand Island, NY, USA) were added at a ratio of 1:10. The inoculated cells were cultured for 3–4 days at 37°C in 5% CO_2_ atmosphere and were blindly passaged 5 times. One field strain of PEDV (named BM1) was successfully adapted for growth on Vero cells. This virus was isolated from a 60-sow farm (identified as BM farm) that had not vaccinated its animals against PEDV. Pigs of all ages from the farm showed clinical symptoms of diarrhea, and death occurred for 100% of suckling piglets and 10% of sows. Examination at necropsy revealed that the dead piglets from BM farm were covered with brown blotches of dried diarrheal feces and their stomachs were filled with undigested milk. Thin, translucent small intestines that contained yellow fluid were also observed (Technical Appendix Figure 1). The BM1 PEDV field isolate induced cytopathic effects of rounded shape (Technical Appendix Figure 2, panel A) within 48 hours at passage 10. The presence of PEDV in the cell culture was confirmed by immunofluorescence assay (VDPro PEDV FA Reagent kit, MEDIAN Diagnostics, Gangwon-do, South Korea), which showed the specific fluorescence signal (Technical Appendix Figure 2, panel B). In addition to evidence by microscopic observation, real-time reverse transcription PCR showed that the quantity of viral RNA increased incrementally as the number of passages increased: from 30,325 copies/μL (cycle threshold 16.11) at passage 2 to 418,000 copies/μL (cycle threshold 13.77) at passage 10. Infective titers of the BM1 isolate increased from 10^4.7^ 50% tissue culture infectious doses/mL at passage 2 to 10^7.9^ 50% tissue culture infectious doses/mL at passage 10 (Technical Appendix; Technical Appendix Table 2).

The complete S gene of BM1 (GenBank accession no. KP861982) was sequenced for genetic characterization; the gene was 4,161-nt long and encoded 1,386 aa. The spike protein of the BM1 isolate showed substitutions at neutralizing SS6 epitope from LQDGQVKI (*7*) to SQSGQVKI but identity at the SS2 (*7*) and 2C10 (*8*) neutralizing epitopes. The genetic relationship of the BM1 isolate with other PEDVs in the world was inferred from a codon-based alignment of 409 sequences of the complete S gene (Technical Appendix Table 3). The maximum-likelihood phylogenetic tree was constructed by using the FastTree program (*9*), with the general time reversible nucleotide substitution model. The phylogeny constructed on the basis of the complete S gene (Figure) showed that the BM1 isolate belongs to subgroup 2a, genogroup 2 of PEDV. This isolate clustered closely with emergent PEDV strains in the United States (Technical Appendix Figure 3), showing 99.2%–99.7% identity with PEDVs of North American strains (*10*). This observation was repeated by the phylogenetic inference of the complete N gene (Figure; Technical Appendix Table 4 and Figure 4). The branching pattern (Figure) clearly showed that BM1 is genetically less related (92.9–93.4% identity) to the live vaccine strains that are derived from genogroup 1 and used currently to prevent PEDV infections in South Korea.

**Figure:**
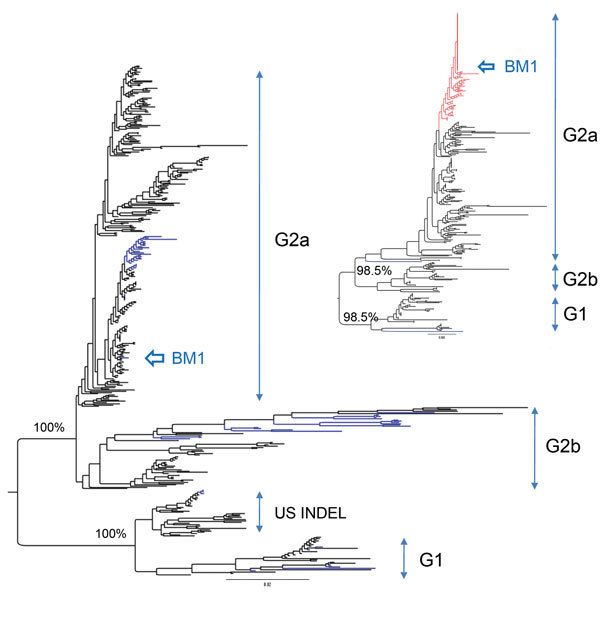
Maximum-likelihood phylogenetic tree of porcine epidemic diarrhea virus from piglet, South Korea, 2013–2014, constructed on the basis of codon alignment of complete S genes. Inset shows a phylogenetic tree inferred from the complete N genes. Genogroups are shown to the right of each tree. US INDEL is a prototype strain of porcine epidemic diarrhea virus that has insertions and deletions (INDELS) in the spike gene. Scale bars indicate nucleotide substitutions per site.

In summary, we isolated the BM1 strain (GenBank accession no. KP861982) in South Korea from a sample from a suckling pig with severe diarrhea; the pig came from a farm that had not vaccinated its pigs against PEDV. The strain was adapted and grew to high titers on Vero cells. The isolate belongs to genogroup 2 and genetically clustered with emerging PEDVs of North American strains but was loosely related to genogroup 1, the basis of the vaccine used for inoculation against Korean PEDV strains. This isolate may need further evaluation as a candidate for a vaccine to prevent reemerging PEDVs in South Korea.

**Technical Appendix.** Detailed methods and experimental findings for isolation of porcine epidemic diarrhea virus during outbreaks in South Korea, 2014.
