# Supplementary material for: Isolation of Porcine Epidemic Diarrhea Virus during Outbreaks in South Korea, 2013–2014
Source: Emerg Infect Dis. 2015 Dec;21(12):2238–40. doi: 10.3201/eid2112.150437 (PMC4672425; doi:10.3201/eid2112.150437)
Supplement: Supplementary file 1 — Technical Appendix. Detailed methods and experimental findings for isolation of porcine epidemic diarrhea virus during outbreaks in South Korea, 2014. [file 15-0437-Techapp-s1.pdf]

Article DOI: http://dx.doi.org/10.3201/eid2112.150437

# Isolation of Porcine Epidemic Diarrhea Virus during Outbreaks in South Korea, 2013–2014

## Technical Appendix

## Methods, Tables, and Figures Related to Phylogenetic Characterization of Porcine Epidemic Diarrhea Virus Isolate

### RNA Extraction and RT-PCR

Total RNA was extracted by using RNA mini kit (Qiagen, Valencia, CA, USA), following the manufacturer's instructions. The RNA was then converted into cDNA with the use of random hexamers and commercial M-MLV reverse transcription kit (Invitrogen, Carlsbad, CA, USA), following manufacturer's protocol. Finally, PCR reactions were performed with pathogen-specific primers by using AccuPower ProFi Taq PCR PreMix (Bioneer Ltd, Daejeon, South Korea). The specific primers for detecting porcine epidemic diarrhea virus (PEDV) were PEDV-420F [5′- TAATTGCCCTTTCACCTTGC –3′] and PEDV-420R [5′- TCAACATATGCAGCCTGCTC –3′], which amplified the 420-bp region of the conserved spike coding protein 2 portion (S2) gene (*1*,*2*). The thermal profile was initial denaturation at 94°C for 5 min, followed by 38 cycles of 94°C for 30 s, 56°C for 45 s, 72°C for 45 s, and a final extension at 72°C for 7min.

### Real-time Quantitative Reverse Transcription PCR

Real-time reverse transcription PCR (Maxima SYBR Green kit, Thermo Fisher Scientific Inc., Grand Island, NY, USA) was performed to quantify the viral RNA of each passage. The PEDV-specific primers were designed as PEDV-QF [5′- TCGCTCTGTGGCAGATCTAGTC-3′] and PEDV-QR [5′- GCGTCAACAACGCCAGGTA –3′]. Each of the expression levels of target genes was automatically calculated as $C_T$ values by comparing standard curve values of the PEDV through real-time quantitative reverse transcription PCR (StepOnePlus, Applied Biosystems, Foster City, CA, USA).

### Sequencing of Spike Protein and Nucleoprotein-Coding Genes

In this study, a new set of specific primers was designed on the basis of the reference strain of USA/Colorado/2013, KF272920 to amplify complete S and N genes of PEDV. Technical Appendix Table 1 shows the sequences of primers and the annealing temperature of PCR.

### Gel Extraction and TA Cloning

The specific PCR bands, which were purified by using the QIAquick Gel Extraction Kit (Qiagen, Düsseldorf, Germany), were used for cloning with the TA cloning kit (Topcloner TA kit, Enzynomics, Daejeon, South Korea) and subsequently transformed into competent *Escherichia coli* cells (DH5α). The purified DNAs were sequenced by using Macrogen sequencer (Seoul, South Korea) in both directions to verify the accuracy of the sequence (*3*).

### References


1. Song D, Park B. Porcine epidemic diarrhoea virus: a comprehensive review of molecular epidemiology, diagnosis, and vaccines. Virus Genes. 2012;44:167–75. http://dx.doi.org/10.1007/s11262-012-0713-1

2. Choi JC, Lee K, Pi J, Park S, Song C, Choi I, et al. Comparative genome analysis and molecular epidemiology of the reemerging porcine epidemic diarrhea virus strains isolated in Korea. Infect Genet Evol. 2014;26:348–51. http://dx.doi.org/10.1016/j.meegid.2014.06.005

3. Kim AR, Chung HC, Kim HK, Kim EO, Choi MG, Yang HJ, et al. Characterization of a complete genome of a circular single-stranded DNA virus from porcine stools in Korea. Virus Genes. 2014;48:81–8. http://dx.doi.org/10.1007/s11262-013-1003-2

4. Huang YW, Dickerman AW, Pineyro P, Li L, Fang L, Kiehne R, et al. Origin, evolution, and genotyping of emergent porcine epidemic diarrhea virus strains in the United States. Mbio. 2013;4:e00737.

5. Wang S, Cheng X, Chen S, Lin F, Jiang B, Zhu X, et al. Classification of emergent U.S. strains of porcine epidemic diarrhea virus by phylogenetic analysis of nucleocapsid and ORF3 genes. J ClinMicrobiol. 2014;52:3509–10. http://dx.doi.org/10.1128/JCM.01708-14

**Technical Appendix Table 1.** Sequences of primers and the annealing temperature of PCR.

| Target gene | Primer name | Sequence (5′-3′) | Product size (bp) | Annealing temperature (°C) | Position |
|---|---|---|---|---|---|
| S | FuS1F | TCTTCTGGCGTAATTCCACA | 937 | 53.3 | 20420–20439 |
| | FuS1R | ATACATTGGCRGCRTAACCA | | | 21338–21357 |
| S | FuS2F | GYGTTGCGACAARRTGTTAC | 993 | 50.3 | 21205–21224 |
| | FuS2R | CAAARGMMGCAGAGACAGTA | | | 22178–22197 |
| S | SF1 | TCATCCATTAGTGATGTTGTGTTA | 1666 | 52.9 | 20527–20550 |
| | SR1 | GCCGCAGAGACAGTAATATTAACA | | | 22169–22192 |
| S | SF2 | GTGTTCTCAGGTTGCTTTTGACCT | 1593 | 59.0 | 22049–22072 |
| | SR2 | AAAGACTCAGCAAGCAATTGCTGG | | | 23618–23641 |
| S | SF3 | GTACAGTGCGTCTCTCATAGGTGG | 1422 | 53.9 | 23489–23512 |
| | SR3 | TCTAATTGGAACTACATTGAGCTC | | | 24887–24910 |
| N | NF1 | TTATGGCTTCTGTCAGTTTT | 1544 | 57.4 | 26377–26396 |
| | NF2 | AGATGAAAAGGTACTGCGTTCC | | | 27899–27920 |

**Technical Appendix Table 2.** Titration of BM1 PEDV isolate from passage 1 to passage 10, South Korea, 2013–2014*

| BM1 sample parameter | Intestine† | P1 | P2 | P3 | P4 | P5 | P6 | P7 | P8 | P9 | P10 |
|---|---|---|---|---|---|---|---|---|---|---|---|
| Cytopathic effect | ND | ND | + | + | + | + | + | + | + | + | + |
| RT-PCR | + | + | + | + | + | + | + | + | + | + | + |
| Real-time (copies/µl; $C_T$ value) | 1,412; 17.96 | 1,905; 17.91 | 30,325; 16.11 | 21,633; 16.42 | 24,727; 16.3 | 7,287; 17.39 | 16,565; 16.77 | 33,905; 16.02 | 80,579; 15.24 | 283,000; 13.98 | 418,000; 13.77 |
| Infectious titer ($\log_{10}$ $TCID_{50}$/ml) | ND | ND | 4.7 | 4.2 | 5.2 | 2.7 | 5.2 | 5.7 | 6.2 | 7.2 | 7.9 |

*ND, not determined; P, passage; PEDV, porcine epidemic diarrhea virus; RT-PCR, reverse transcription PCR; +, positive.
†0.02 µm filtered of 10% intestine homogenized suspension.

**Technical Appendix Table 3.** List of complete spike protein-coding sequences used in PEDV study, South Korea, 2013–2014*

| GenBank accession no. | Strain name | Country | Genogroup | Reference |
|---|---|---|---|---|
| Z25483 | Br1/87 | | | |
| AF353511 | CV777 | | 1a | (*4*) |
| AF500215 | Korea | | | |
| AY167585 | Chinju99 | | | |
| AY653204 | JS20042 | China | | |
| DQ462404 | DR13 | Korea | | |
| DQ862099 | DR13 | Korea | | |
| DQ985739 | LJB/03 | China | | |
| EU031893 | DX | China | | |
| GU180142 | KNU0801 | Korea | | |
| GU180143 | KNU0802 | Korea | | |
| GU180144 | KNU0901 | Korea | | |
| GU180145 | KNU0902 | Korea | | |
| GU180146 | KNU0903 | Korea | | |
| GU180147 | KNU0904 | Korea | | |
| GU180148 | KNU0905 | Korea | | |
| AB548618 | 83P5 | Japan | | |
| AB548619 | 83P5 | Japan | | |
| AB548620 | 83P5 | Japan | | |
| AB548621 | 83P5 | Japan | | |
| AB548622 | KH | Japan | | |
| AB548623 | NK | Japan | | |
| AB548624 | MK | Japan | | |
| JN315706 | CH/FJND2/2011 | China | | |
| JN381492 | CH/FJND3/2011 | China | | |
| JN543367 | CH/FJND1/2011 | China | | |
| JN184634 | CNU09122201 | Korea | | |
| JN184635 | CNU09122202 | Korea | | |
| JN980698 | CHGD01 | China | | |
| JQ257005 | CH/KF/11 | China | | |
| JQ257006 | CH/YY/11 | China | | |
| JQ257007 | CH/ZY/11 | China | | |
| JN599150 | CV777 | China | | |

| GenBank accession no. | Strain name | Country | Genogroup | Reference |
|---|---|---|---|---|
| JN825706 | BJ20112 | China | | |
| JN825707 | HB20111 | China | | |
| JN825708 | HB20112 | China | | |
| JN825709 | HB20113 | China | | |
| JN825710 | ZJ20111 | China | | |
| JN825711 | ZJ20112 | China | | |
| JN825712 | BJ20111 | China | 2a | (*4*) |
| JQ517274 | HuN | China | | |
| JQ627653 | CH/AY/11 | China | | |
| JQ627654 | CH/CG/11 | China | | |
| JQ638915 | CH/GD/2011 | China | | |
| JQ638916 | CH/HLJHH/2011 | China | | |
| JQ638917 | CH/SDLY/2011 | China | | |
| JQ638918 | CH/HBBD/2011 | China | | |
| JQ638919 | CH/SDQD/2011 | China | | |
| JQ638920 | CH/JLCC/2011 | China | | |
| JQ638921 | CH/BJSY/2011 | China | | |
| JQ638922 | CH/HBQHD/2011 | China | | |
| JQ638923 | CH/JLGZL/2011 | China | | |
| JQ638924 | CH/JL/2011 | China | | |
| JQ239429 | CH1 | China | | |
| JQ239430 | CH2 | China | | |
| JQ239431 | CH3 | China | | |
| JQ239432 | CH4 | China | | |
| JQ239433 | CH5 | China | | |
| JQ239434 | CH6 | China | | |
| JQ239435 | CH7 | China | | |
| JQ239436 | CH8 | China | | |
| JQ979287 | CH9FJ | China | | |
| JQ979288 | CH13GX | China | | |
| JQ979289 | CH17GZ | China | | |
| JQ979290 | CH22JS | China | | |
| JQ979291 | CH18Hainan | China | | |
| JX018179 | CH/GXNN/2012 | China | | |
| JX018180 | CH/YNKM/2012 | China | | |
| JX018181 | CH/AHHF/2012 | China | | |
| JX018182 | CH/AHHF2/2012 | China | | |
| JX018183 | CH/HBSN/2012 | China | | |
| JX070671 | CH/FJXM1/2012 | China | | |
| JX070672 | CH/FJXM2/2012 | China | | |
| JX163294 | HBMC2012 | China | | |
| JX258672 | FQ/FJ/2012 | China | | |
| JX242454 | CHHYC082011 | | | |
| JX242455 | CHHYC102011 | | | |
| JX242456 | CHHYC112011 | | | |
| JX242457 | CHHYC122011 | | | |
| JX242458 | CHLC102011 | | | |
| JX242459 | CHSONGB122011 | | | |
| JX242460 | CHYHC122011 | | | |
| JX242461 | CHZWC122011 | | | |
| JX242462 | CHHKC082011 | | | |
| JX242463 | CHLC122011 | | | |
| JX242464 | CHSHT122011 | | | |
| JX512907 | HLJ2012 | China | | |
| JX435298 | BJ20113 | China | | |
| JX435299 | BJ20121 | China | | |
| JX435300 | BJ20122 | China | | |
| JX435301 | HB20114 | China | | |
| JX435302 | HB20121 | China | | |
| JX435303 | HB20122 | China | | |
| JX435304 | HB20123 | China | | |
| JX435305 | HB20124 | China | | |
| JX489155 | LC | China | 2b | (*4*) |
| JX524137 | ZJCZ4 | China | 2b | (*4*) |
| JX501317 | CH/CY/12 | China | | |
| JX501318 | CH/HBQX/10 | China | | |
| JX501319 | CH/HBXX2/11 | China | | |
| JX501320 | CH/HBXX3/11 | China | | |
| JX501321 | CH/TY/12 | China | | |

| GenBank accession no. | Strain name | Country | Genogroup | Reference |
|---|---|---|---|---|
| JX501322 | CH/XC/12 | China | | |
| JX501323 | CH/XCYL/11 | China | | |
| JX647847 | GD1 | China | 2b | (*4*) |
| JX560761 | SDM | China | 1b | (*4*) |
| KC196276 | CH/ZMDZY/11 | China | 2a | (*4*) |
| KC242897 | FJZZ12011 | China | | |
| KC242898 | HeN1220122 | China | | |
| KC242899 | HB120122 | China | | |
| KC242900 | HB220122 | China | | |
| KC242901 | AH220123 | China | | |
| KC242902 | HB320123 | China | | |
| KC242903 | ZJ1720125 | China | | |
| KC242904 | YN220127 | China | | |
| KC242905 | FJ520128 | China | | |
| KC242906 | FJZZ920128 | China | | |
| KC242907 | JX420129 | China | | |
| KC242908 | HN420129 | China | | |
| KC242909 | GD520129 | China | | |
| KC242910 | GD820129 | China | | |
| KC242911 | HN6201211 | China | | |
| KC242912 | HN8201211 | China | | |
| KC787536 | CHCCC012013 | China | | |
| KC787537 | CHDLC012013 | China | | |
| KC787538 | CHGGC112012 | China | | |
| KC787539 | CHGMB022013 | China | | |
| KC787540 | CHHGC012013 | China | | |
| KC787541 | CHLNC012012 | China | | |
| KC787542 | CHSBC032013 | China | | |
| KC787543 | CHSTC122012 | China | | |
| KC787544 | CHYGC122012 | China | | |
| KC787545 | CHYXC012013 | China | | |
| KF384500 | CH/GDGZ/2012 | China | | |
| KC879275 | AS01 | Korea | | |
| KC879276 | AS02 | Korea | | |
| KC879277 | AS03 | Korea | | |
| KC879278 | NJ01 | Korea | | |
| KC879279 | NJ02 | Korea | | |
| KC879280 | AD01 | Korea | | |
| KC879281 | AD02 | Korea | | |
| KC879282 | AD03 | Korea | | |
| KF453510 | AH120411 | China | | |
| KF453511 | GX120308 | China | | |
| KF453512 | HB121229 | China | | |
| KF453514 | SH120301 | China | | |
| KF453515 | SH121208 | China | | |
| KF453516 | ZJ110618 | China | | |
| KF453517 | ZJ111113 | China | | |
| KC886306 | SCL | China | | |
| KF177254 | JY5C | China | | |
| KF177255 | JY6C | China | | |
| KF177256 | JY7C | China | | |
| KF177257 | YJ3F | China | | |
| KF177258 | YJ7C | China | | |
| KF294254 | HZXS1 | China | | |
| KF294255 | HZXS2 | China | | |
| KF294256 | GJL | China | | |
| KF468752 | MN | USA | 2a | (*4*) |
| KF468753 | IA1 | USA | 2a | (*4*) |
| KF468754 | IA2 | USA | 2a | (*4*) |
| KF468755 | XS2013 | China | | |
| AB857233 | GDS01 | China | | |
| AB857234 | GDS02 | China | | |
| AB857235 | GDS03 | China | | |
| KF546800 | GDFS/2011 | China | | |
| KF546801 | GDQY/2011 | China | | |
| KF546802 | GDDG/2011 | China | | |
| KF546803 | GDJM/2012 | China | | |
| KF546804 | GDZQ/2012 | China | | |
| KF601195 | GXLZ/2013 | China | | |

| GenBank accession no. | Strain name | Country | Genogroup | Reference |
|---|---|---|---|---|
| KF601196 | JXGZ/2013 | China | | |
| KF601197 | HNCZ/2013 | China | | |
| KF601198 | JXJA/2013 | China | | |
| KF601199 | GXHZ/2013 | China | | |
| KF601200 | GDEP/2013 | China | | |
| KF601201 | GXNN/2013 | China | | |
| KF577730 | YNKM/2012 | China | | |
| KF577731 | GDMZ/2013 | China | | |
| KF577732 | GDGZ/2013 | China | | |
| KC764952 | NPPED2008 | Thailand | | |
| KC764953 | NPPED0108 | Thailand | | |
| KC764954 | PED0212 | Thailand | | |
| KC764955 | PED0210 | Thailand | | |
| KC764956 | SBPED0211 | Thailand | | |
| KC764957 | SBPED0211 | Thailand | | |
| KC764958 | SPPED0212 | Thailand | | |
| KC764959 | SBPED0211 | Thailand | | |
| KC764960 | SPPED0212 | Thailand | | |
| KF724935 | 45RWVCF0712 | Thailand | | |
| KF724936 | 155ST0412 | Thailand | | |
| KF724937 | 2253AG0211 | Thailand | | |
| KF724938 | 656ST0413 | Thailand | | |
| KF738258 | GD120330 | China | | |
| KF738259 | JS130604 | China | | |
| KF738260 | JX130506 | China | | |
| KF738261 | SD130502 | China | | |
| KF738262 | SD130611 | China | | |
| KF738263 | SH121229 | China | | |
| KF738264 | ZJ121030 | China | | |
| KJ158152 | AHM | China | | |
| KF779469 | MF3809/2008 | Korea | | |
| KF898124 | KPEDV9 | Korea | | |
| KJ020932 | CHYJ130330 | China | | |
| KJ196348 | SHQP/YM/2013 | China | | |
| KJ588062 | KUIDLPED2014007 | Korea | | |
| KJ588063 | KUIDLPED2014002 | Korea | | |
| KJ588064 | KUIDLPED2014001 | Korea | | |
| KJ451036 | KNU1301 | Korea | | |
| KJ451037 | KNU1302 | Korea | | |
| KJ451038 | KNU1303 | Korea | | |
| KJ451039 | KNU1304 | Korea | | |
| KJ451040 | KNU1305 | Korea | | |
| KJ451041 | KNU1306 | Korea | | |
| KJ451042 | KNU1307 | Korea | | |
| KJ451043 | KNU1308 | Korea | | |
| KJ451044 | KNU1309 | Korea | | |
| KJ451046 | KNU1311 | Korea | | |
| KJ451047 | KNU1401 | Korea | | |
| KJ451048 | KNU1402 | Korea | | |
| KJ451045 | KNU1310 | Korea | | |
| KJ539151 | K13JA121 | Korea | | |
| KJ539152 | K13JA123 | Korea | | |
| KJ539153 | K13JA114 | Korea | | |
| KJ539154 | K14JB01 | Korea | | |
| KJ646578 | FJZP 2013 | China | | |
| KJ646579 | FJCL 2013 | China | | |
| KJ646580 | FJFQ 2014 | China | | |
| KJ646581 | FJFQ1 2012 | China | | |
| KJ646582 | FJFQ2 2012 | China | | |
| KJ646583 | FJLY 2012 | China | | |
| KJ646584 | FJLY 2013 | China | | |
| KJ646585 | FJND 2012 | China | | |
| KJ646586 | FJND 2013 | China | | |
| KJ646587 | FJPT 2013 | China | | |
| KJ646588 | FJQK 2013 | China | | |
| KJ646589 | FJQZ 2013 | China | | |
| KJ646590 | FJYX 2013 | China | | |
| KJ646591 | FJZP 2014 | China | | |
| KJ642641 | NJ | China | | |

| GenBank accession no. | Strain name | Country | Genogroup | Reference |
|---|---|---|---|---|
| KJ645635 | Indiana12.83/2013 | USA | | |
| KJ645636 | Iowa28/2013 | USA | | |
| KJ645637 | Kansas29/2013 | USA | | |
| KJ645638 | Colorado30/2013 | USA | | |
| KJ645639 | Texas31/2013 | USA | | |
| KJ645640 | Oklahoma32/2013 | USA | | |
| KJ645641 | Indiana34/2013 | USA | | |
| KJ645642 | Oklahoma35/2013 | USA | | |
| KJ645643 | Kansas36/2013 | USA | | |
| KJ645644 | Oklahoma38/2013 | USA | | |
| KJ645645 | Texas39/2013 | USA | | |
| KJ645646 | NorthCarolina40/2013 | USA | | |
| KJ645647 | Minnesota41/2013 | USA | | |
| KJ645648 | Minnesota42/2013 | USA | | |
| KJ645649 | Iowa23.57/2013 | USA | | |
| KJ645650 | Kansas46/2013 | USA | | |
| KJ645651 | Colorado47/2013 | USA | | |
| KJ645652 | Minnesota54/2013 | USA | | |
| KJ645653 | Wisconsin55/2013 | USA | | |
| KJ645654 | Tennesse56/2013 | USA | | |
| KJ645655 | Minnesota58/2013 | USA | | |
| KJ645656 | Minnesota59/2013 | USA | | |
| KJ645657 | Ohio60/2013 | USA | | |
| KJ645658 | Minnesota62/2013 | USA | | |
| KJ645659 | Illinois63/2013 | USA | | |
| KJ645660 | Minnesota64/2013 | USA | | |
| KJ645661 | Minnesota65/2013 | USA | | |
| KJ645662 | NorthCarolina66/2013 | USA | | |
| KJ645663 | Minnesota67/2013 | USA | | |
| KJ645664 | Ohio68/2013 | USA | | |
| KJ645665 | Ohio69/2013 | USA | | |
| KJ645666 | Iowa70/2013 | USA | | |
| KJ645667 | Minnesota72/2013 | USA | | |
| KJ645668 | Minnesota73/2013 | USA | | |
| KJ645669 | Wisconsin74/2013 | USA | | |
| KJ645670 | Ohio75/2013 | USA | | |
| KJ645671 | Minnesota76/2013 | USA | | |
| KJ645672 | Minnesota77/2013 | USA | | |
| KJ645673 | Minnesota78/2013 | USA | | |
| KJ645674 | Minnesota79/2013 | USA | | |
| KJ645675 | Illinois81/2013 | USA | | |
| KJ645676 | Minnesota82/2013 | USA | | |
| KJ645677 | Minnesota83/2013 | USA | | |
| KJ645678 | Minnesota85/2013 | USA | | |
| KJ645679 | Minnesota86/2013 | USA | | |
| KJ645680 | Illinois87/2013 | USA | | |
| KJ645681 | Minnesota89/2013 | USA | | |
| KJ645682 | Minnesota90/2013 | USA | | |
| KJ645683 | NorthCarolina91/2013 | USA | | |
| KJ645684 | Missouri92/2013 | USA | | |
| KJ645685 | Missouri93/2013 | USA | | |
| KJ645686 | Minnesota94/2013 | USA | | |
| KJ645687 | Minnesota95/2013 | USA | | |
| KJ645688 | Iowa96/2013 | USA | | |
| KJ645689 | Illinois97/2013 | USA | | |
| KJ645690 | Illinois98/2013 | USA | | |
| KJ645691 | Minnesota100/2013 | USA | | |
| KJ645692 | Missouri101/2013 | USA | | |
| KJ645693 | Missouri102/2013 | USA | | |
| KJ645694 | Iowa103/2013 | USA | | |
| KJ645695 | Iowa106/2013 | USA | | |
| KJ645696 | Iowa107/2013 | USA | | |
| KJ645697 | Texas128/2014 | USA | | |
| KJ645698 | Ohio120/2014 | USA | | |
| KJ645699 | Ohio123/2014 | USA | | |
| KJ645700 | MEX/124/2014 | Mexico | | |
| KJ645701 | Kansas125/2014 | USA | | |
| KJ645702 | Ohio126/2014 | USA | | |
| KJ645703 | Minnesota127/2014 | USA | | |

| GenBank accession no. | Strain name | Country | Genogroup | Reference |
|---|---|---|---|---|
| KJ645704 | Minnesota52/2013 | USA | | |
| KJ645705 | Minnesota61/2013 | USA | | |
| KJ645706 | Minnesota71/2013 | USA | | |
| KJ645707 | Minnesota84/2013 | USA | | |
| KJ645708 | MEX/104/2013 | Mexico | | |
| KJ662670 | KNU1305 | Korea | | |
| KF840537 | CH/ZJCX1/2012 | China | | |
| KF840538 | CH/ZJHY2/2012 | China | | |
| KF840539 | CH/ZJJSZ/2012 | China | | |
| KF840540 | CH/ZJJS1Z/2012 | China | | |
| KF840541 | CH/JXZS3H/2012 | China | | |
| KF840542 | CH/JXZS2H/2012 | China | | |
| KF840543 | CH/ZJXS212/2012 | China | | |
| KF840544 | CH/ZJHZHY6/2013 | China | | |
| KF840545 | CH/JXJDZF/2012 | China | | |
| KF840546 | CH/ZJJS2Z/2012 | China | | |
| KF840547 | CH/JXZS3L | China | | |
| KF840548 | CH/JXZS1223L | China | | |
| KF840549 | CH/SDZD1/2012 | China | | |
| KF840550 | CH/SDZD2/2012 | China | | |
| KF840551 | CH/HuBWHYQ/2012 | China | | |
| KF840552 | CH/ZJQZ2/2012 | China | | |
| KF840553 | CH/ZJHZ1C/2012 | China | | |
| KF840554 | CH/ZJHZ2C/2012 | China | | |
| KF840555 | CH/JXJDZ1/2012 | China | | |
| KF840556 | CH/ZJJS4X/2012 | China | | |
| KF840561 | CH/JSZLS2/2013 | China | | |
| KF840562 | CH/JSZLN2/2013 | China | | |
| KJ741221 | KNU14091 | Korea | | |
| KJ741222 | KNU14092 | Korea | | |
| KJ741223 | KNU14093 | Korea | | |
| KJ741224 | KNU14094 | Korea | | |
| KJ741225 | KNU14095 | Korea | | |
| KJ767195 | OhioVBS1 | USA | | |
| KJ767196 | OhioVBS2 | USA | | |
| KJ777677 | LZW | China | | |
| KJ777678 | LZW | China | | |
| KM189366 | ON007 | Canada | | |
| KM189368 | PEI023 | Canada | | |
| KM196109 | SK030 | Canada | | |
| KM196110 | 2014022 | Canada | | |
| KM196111 | MB021 | Canada | | |
| KM077139 | Minnesota188/2014 | USA | | |
| KM392224 | TC PC22AP10 | USA | | |
| KM392226 | TC PC168P2 | USA | | |
| KM392231 | TC PC182P2 | USA | | |
| KM392232 | TC Iowa106 (PV39)P1 | USA | | |
| KM392225 | TC PE103(PC21A)P4 | USA | | |
| KM392227 | TC PC170P2 | USA | | |
| KM392228 | TCPC173P2 | USA | | |
| KM392229 | TC PC177P2 | USA | | |
| KM392230 | TC PC180P2 | USA | | |
| KM403155 | KNU14061 | Korea | | |
| KM403156 | KNU14062 | Korea | | |
| KM403157 | KNU14063 | Korea | | |
| KM403158 | KNU14064 | Korea | | |
| KM108348 | KPV1401 | Korea | | |
| KM108349 | KPV1402 | Korea | | |
| KM108350 | KPV1403 | Korea | | |
| KM108351 | KPV1404 | Korea | | |
| KM108352 | KPV1405 | Korea | | |
| KM108353 | KPV1406 | Korea | | |
| KM242131 | CH/GDZQ/2014 | China | | |
| KM287429 | XJDB2 | China | | |
| KM406178 | LS1401 | China | | |
| KM406179 | MY1402 | China | | |
| KM406180 | DY1403 | China | | |
| KM406181 | SH1404 | China | | |
| KM406182 | MX1405 | China | | |

| GenBank accession no. | Strain name | Country | Genogroup | Reference |
|---|---|---|---|---|
| KM406183 | PZ1406 | China | | |
| KM406184 | PJ1407 | China | | |
| KP455313 | HUAPED45 | Vietnam | | |
| KP455314 | HUAPED47 | Vietnam | | |
| KP455315 | HUAPED55 | Vietnam | | |
| KP455316 | HUAPED58 | Vietnam | | |
| KP455317 | HUAPED60 | Vietnam | | |
| KP455318 | HUAPED63 | Vietnam | | |
| KP455319 | HUAPED67 | Vietnam | | |
| KP455320 | HUAPED68 | Vietnam | | |
| KP861982(**PEDV)** | **BM1** | **Korea** | | **This study** |
| JQ771753 | YS | China | | |
| JQ771752 | ZB | China | | |
| JQ771751 | SH | China | | |
| KJ399978 | OH851 | USA | | |
| KC210145 | | China | 2a | |
| JX088695 | | China | 2a | |
| KC210147 | | China | 2a | |
| JQ282909 | | China | 2a | |
| KC140102 | | China | 2a | |
| JX188454 | | China | 2b | |
| JX261936 | | China | 2b | |
| JX112709 | | China | 2b | |
| JQ023161 | vDR13 | Korea | 1r | (*4*) |
| JN547228 | | China | 1r | |
| EF185992 | | China | 1a | |
| GU937797 | | Korea | 1a | |
| KC109141 | | China | 1b | |
| KC210146 | | China | 1b | |
| JQ023162 | aDR13 | Korea | 1b | (*4*) |
| KC189944 | | China | 1b | |

*Information about strain name and country were extracted from Genbank. For some entries, the corresponding information was not available. Genogroups for some strains were obtained from the cited reference. PEDV, porcine epidemic diarrhea virus.

**Technical Appendix Table 4**. List of complete nucleoprotein-coding sequences used in PEDV study, South Korea, 2013–2014*

| GenBank accession no. | Strain name | Country | Genogroup | Reference |
|---|---|---|---|---|
| DQ355224 | | China | | |
| DQ355223 | | China | | |
| DQ355221 | | China | | |
| JN601062 | | China | G2.1 | (*5*) |
| JN601061 | | China | | |
| JN601060 | | China | | |
| JN601059 | | China | G1 | (*5*) |
| JN601058 | | China | G2.3 | (*5*) |
| JN601057 | | China | G2.3 | (*5*) |
| JN601056 | | China | G1 | (*5*) |
| JN601055 | | China | G2.1 | (*5*) |
| JN601054 | | China | | |
| JN601053 | | China | G1 | (*5*) |
| JN601052 | | China | | |
| KR003452 | | Belgium | | |
| KM609213 | | China | | |
| KM609212 | | China | | |
| KM609211 | | China | | |
| KM609210 | | China | | |
| KM609209 | | China | | |
| KM609208 | | China | | |
| KM609207 | | China | | |
| KM609206 | | China | | |
| KM609205 | | China | | |
| KM609204 | | China | | |
| KM609203 | | China | | |
| NC | 003436 | | | |
| KM242131 | CH/GDZQ/2014 | China | | |
| KM403155 | KNU14061 | Korea | | |
| KF840558 | | China | | |
| KF840557 | | China | | |

| GenBank accession no. | Strain name | Country | Genogroup | Reference |
|---|---|---|---|---|
| KF840556 | CH/ZJJS4X/2012 | China | | |
| KF840555 | CH/JXJDZ1/2012 | China | | |
| KF840554 | CH/ZJHZ2C/2012 | China | | |
| KF840553 | CH/ZJHZ1C/2012 | China | | |
| KF840552 | CH/ZJQZ2/2012 | China | | |
| KF840551 | CH/HuBWHYQ/2012 | China | | |
| KF840550 | CH/SDZD2/2012 | China | | |
| KF840549 | CH/SDZD1/2012 | China | | |
| KF840548 | CH/JXZS1223L | China | | |
| KF840547 | CH/JXZS3L | China | | |
| KF840546 | CH/ZJJS2Z/2012 | China | | |
| KF840545 | CH/JXJDZF/2012 | China | | |
| KF840544 | CH/ZJHZHY6/2013 | China | | |
| KF840543 | CH/ZJXS212/2012 | China | | |
| KF840542 | CH/JXZS2H/2012 | China | | |
| KF840541 | CH/JXZS3H/2012 | China | | |
| KF840540 | CH/ZJJS1Z/2012 | China | | |
| KF840539 | CH/ZJJSZ/2012 | China | | |
| KF840538 | CH/ZJHY2/2012 | China | | |
| KF840537 | CH/ZJCX1/2012 | China | | |
| KJ196348 | SHQP/YM/2013 | China | | |
| KM392230 | TCPC180P2 | USA | | |
| KM392229 | TCPC177P2 | USA | | |
| KM392228 | TCPC173P2 | USA | | |
| KM392227 | TCPC170P2 | USA | | |
| KM392225 | TCPE103(PC21A)P4 | USA | | |
| KM392232 | TCIowa106 (PV39)P1 | USA | | |
| KM392231 | TCPC182P2 | USA | | |
| KM392226 | TCPC168P2 | USA | | |
| KM392224 | TCPC22AP10 | USA | | |
| KJ184549 | | USA | | |
| KJ588064 | KUIDLPED2014001 | Korea | | |
| KJ588063 | KUIDLPED2014002 | Korea | | |
| KJ588062 | KUIDLPED2014007 | Korea | | |
| KM077139 | Minnesota188/2014 | USA | | |
| KJ960180 | | Vietnam | | |
| KJ960179 | | Vietnam | | |
| KJ960178 | | Vietnam | | |
| KJ777678 | LZW | China | | |
| KJ777677 | LZW | China | | |
| KJ662670 | KNU1305 | Korea | | |
| KJ158152 | AHM | China | | |
| KJ642642 | | China | | |
| KJ778616 | | USA | | |
| KJ778615 | | USA | | |
| KJ623926 | | Korea | | |
| KJ646625 | | China | | |
| KJ646624 | | China | | |
| KJ646623 | | China | | |
| KJ646622 | | China | | |
| KJ646621 | | China | | |
| KJ646620 | | China | | |
| KJ646619 | | China | | |
| KJ646618 | | China | | |
| KJ646617 | | China | | |
| KJ646616 | | China | | |
| KJ646615 | | China | | |
| KJ646614 | | China | | |
| KJ646613 | | China | | |
| KJ646612 | | China | | |
| KJ646611 | | China | | |
| KJ646610 | | China | | |
| KJ408801 | | USA | G2.1 | (*5*) |
| KF761675 | | China | | |
| KJ020932 | CHYJ130330 | China | | |
| KJ399978 | OH851 | USA | | |
| KF994808 | | China | | |
| KF994807 | | China | | |
| KF994806 | | China | | |

| GenBank accession no. | Strain name | Country | Genogroup | Reference |
|---|---|---|---|---|
| KF994805 | | China | | |
| KF994804 | | China | | |
| KF994803 | | China | | |
| KF994802 | | China | | |
| KF994801 | | China | | |
| KF994800 | | China | | |
| KF994799 | | China | | |
| KF994798 | | China | | |
| KF994797 | | China | | |
| KF994796 | | China | | |
| KF994795 | | China | | |
| KF994794 | | China | | |
| KF994793 | | China | | |
| KF994792 | | China | | |
| KF994791 | | China | | |
| KF994790 | | China | | |
| KF994789 | | China | | |
| KF804028 | | USA | | |
| KF452323 | | USA | G2.1 | (*5*) |
| KF452322 | | USA | G2.1 | (*5*) |
| KF468752 | MN | USA | 2a | (*4*) |
| KF468754 | IA2 | USA | 2a | (*4*) |
| KF468753 | IA1 | USA | 2a | (*4*) |
| KF384500 | CH/GDGZ/2012 | China | | |
| KF272920 | | USA | G2.1 | (*5*) |
| AB618622 | | Japan | G1 | (*5*) |
| AB618621 | | Japan | | |
| AB618620 | | Japan | | |
| AB618619 | | Japan | G1 | (*5*) |
| KC189944 | | China | 1b | (*4*) |
| JQ735953 | | China | G2.1 | (*5*) |
| JQ743656 | | China | G2.3 | (*5*) |
| JQ743655 | | China | G1 | (*5*) |
| JQ743654 | | China | G2.1 | (*5*) |
| JQ743653 | | China | G1 | (*5*) |
| JQ743652 | | China | G2.1 | (*5*) |
| JQ743651 | | China | G1 | (*5*) |
| JQ743650 | | China | G2.1 | (*5*) |
| HQ455346 | | China | | |
| HQ455345 | | China | G1 | (*5*) |
| KC140102 | | China | 2a | (*4*) |
| KC109141 | | China | 1b | (*4*) |
| JN825712 | BJ20111 | China | 2a | (*4*) |
| KC196276 | CH/ZMDZY/11 | China | 2a | (*4*) |
| JX560761 | SDM | China | 1b | (*4*) |
| JX647847 | GD1 | China | 2b | (*4*) |
| JX524137 | ZJCZ4 | China | 2b | (*4*) |
| JX489155 | LC | China | 2b | (*4*) |
| JX406145 | | China | | |
| JX406144 | | China | | |
| JX406143 | | China | | |
| JX406142 | | China | | |
| JX406141 | | China | | |
| JX406140 | | China | | |
| JX406139 | | China | | |
| JX406138 | | China | | |
| JX406137 | | China | | |
| JX406136 | | China | | |
| JX406135 | | China | | |
| JX406134 | | China | | |
| JX512909 | | China | | |
| JQ723738 | | China | | |
| JQ723737 | | China | | |
| JQ723731 | | China | | |
| JX188454 | | China | 2b | (*4*) |
| JX112709 | | China | 2b | (*4*) |
| JX088695 | | China | 2a | (*4*) |
| JQ023162 | aDR13 | Korea | 1b | (*4*) |
| JQ023161 | vDR13 | Korea | 1r | (*4*) |

| GenBank accession no. | Strain name | Country | Genogroup | Reference |
|---|---|---|---|---|
| JQ282909 | | China | 2a | (*4*) |
| JN547228 | | China | 1r | (*4*) |
| GU937797 | | Korea | 1a | (*4*) |
| AY653206 | | China | G2.1 | (*5*) |
| AF353511 | CV777 | | 1a | (*4*) |
| AF237764 | Korea | | G1 | (*5*) |
| EF185992 | China | | 1a | (*4*) |
| KP691458 | | China | | |
| KJ526096 | | China | | |
| KP202365 | | USA | | |
| KF760557 | | China | | |
| KP162057 | | China | | |
| KM887144 | | China | | |
| KM604665 | | China | | |
| LM645057 | | Germany | | |
| KJ645708 | MEX/104/2013 | Mexico | | |
| KJ645707 | Minnesota84/2013 | USA | | |
| KJ645706 | Minnesota71/2013 | USA | | |
| KJ645705 | Minnesota61/2013 | USA | | |
| KJ645704 | Minnesota52/2013 | USA | | |
| KJ645703 | Minnesota127/2014 | USA | | |
| KJ645702 | Ohio126/2014 | USA | | |
| KJ645701 | Kansas125/2014 | USA | | |
| KJ645700 | MEX/124/2014 | Mexico | | |
| KJ645699 | Ohio123/2014 | USA | | |
| KJ645698 | Ohio120/2014 | USA | | |
| KJ645697 | Texas128/2014 | USA | | |
| KJ645696 | Iowa107/2013 | USA | | |
| KJ645695 | Iowa106/2013 | USA | | |
| KJ645694 | Iowa103/2013 | USA | | |
| KJ645693 | Missouri102/2013 | USA | | |
| KJ645692 | Missouri101/2013 | USA | | |
| KJ645691 | Minnesota100/2013 | USA | | |
| KJ645690 | Illinois98/2013 | USA | | |
| KJ645689 | Illinois97/2013 | USA | | |
| KJ645688 | Iowa96/2013 | USA | | |
| KJ645687 | Minnesota95/2013 | USA | | |
| KJ645686 | Minnesota94/2013 | USA | | |
| KJ645685 | Missouri93/2013 | USA | | |
| KJ645684 | Missouri92/2013 | USA | | |
| KJ645683 | NorthCarolina91/2013 | USA | | |
| KJ645682 | Minnesota90/2013 | USA | | |
| KJ645681 | Minnesota89/2013 | USA | | |
| KJ645680 | Illinois87/2013 | USA | | |
| KJ645679 | Minnesota86/2013 | USA | | |
| KJ645678 | Minnesota85/2013 | USA | | |
| KJ645677 | Minnesota83/2013 | USA | | |
| KJ645676 | Minnesota82/2013 | USA | | |
| KJ645675 | Illinois81/2013 | USA | | |
| KJ645674 | Minnesota79/2013 | USA | | |
| KJ645673 | Minnesota78/2013 | USA | | |
| KJ645672 | Minnesota77/2013 | USA | | |
| KJ645671 | Minnesota76/2013 | USA | | |
| KJ645670 | Ohio75/2013 | USA | | |
| KJ645669 | Wisconsin74/2013 | USA | | |
| KJ645668 | Minnesota73/2013 | USA | | |
| KJ645667 | Minnesota72/2013 | USA | | |
| KJ645666 | Iowa70/2013 | USA | | |
| KJ645665 | Ohio69/2013 | USA | | |
| KJ645664 | Ohio68/2013 | USA | | |
| KJ645663 | Minnesota67/2013 | USA | | |
| KJ645662 | NorthCarolina66/2013 | USA | | |
| KJ645661 | Minnesota65/2013 | USA | | |
| KJ645660 | Minnesota64/2013 | USA | | |
| KJ645659 | Illinois63/2013 | USA | | |
| KJ645658 | Minnesota62/2013 | USA | | |
| KJ645657 | Ohio60/2013 | USA | | |
| KJ645656 | Minnesota59/2013 | USA | | |
| KJ645655 | Minnesota58/2013 | USA | | |

| GenBank accession no. | Strain name | Country | Genogroup | Reference |
|---|---|---|---|---|
| KJ645654 | Tennesse56/2013 | USA | | |
| KJ645653 | Wisconsin55/2013 | USA | | |
| KJ645652 | Minnesota54/2013 | USA | | |
| KJ645651 | Colorado47/2013 | USA | | |
| KJ645650 | Kansas46/2013 | USA | | |
| KJ645649 | Iowa23.57/2013 | USA | | |
| KJ645648 | Minnesota42/2013 | USA | | |
| KJ645647 | Minnesota41/2013 | USA | | |
| KJ645646 | NorthCarolina40/2013 | USA | | |
| KJ645645 | Texas39/2013 | USA | | |
| KJ645644 | Oklahoma38/2013 | USA | | |
| KJ645643 | Kansas36/2013 | USA | | |
| KJ645642 | Oklahoma35/2013 | USA | | |
| KJ645641 | Indiana34/2013 | USA | | |
| KJ645640 | Oklahoma32/2013 | USA | | |
| KJ645639 | Texas31/2013 | USA | | |
| KJ645638 | Colorado30/2013 | USA | | |
| KJ645637 | Kansas29/2013 | USA | | |
| KJ645636 | Iowa28/2013 | USA | | |
| KJ645635 | Indiana12.83/2013 | USA | | |
| KM189367 | | Canada | | |
| KM052365 | | USA | | |
| KJ584361 | | USA | | |
| KF650375 | | USA | G2.1 | (*5*) |
| KF650374 | | USA | G2.1 | (*5*) |
| KF650373 | | USA | G2.1 | (*5*) |
| KF650372 | | USA | G2.1 | (*5*) |
| KF650371 | | USA | G2.1 | (*5*) |
| KF650370 | | USA | G2.1 | (*5*) |
| KF267450 | | USA | G2.1 | (*5*) |
| KC210147 | | China | 2a | (*4*) |
| KC210146 | | China | 1b | (*4*) |
| KC210145 | | China | 2a | (*4*) |
| KC243782 | | China | | |
| JX261936 | | China | 2b | (*4*) |
| JQ081280 | | China | | |
| JQ081279 | | China | | |
| JQ081278 | | China | | |
| JQ081277 | | China | | |
| JQ081276 | | China | | |
| JQ081275 | | China | | |
| JQ081274 | | China | | |
| JQ081273 | | China | | |
| JN255988 | | China | | |
| JN255987 | | China | | |
| JN255986 | | China | | |
| JN255985 | | China | | |
| JN255984 | | China | | |
| JN255983 | | China | | |
| JN255982 | | China | | |
| JN255981 | | China | | |
| JN255980 | | China | | |
| JN255979 | | China | | |
| JN255978 | | China | | |
| JN255977 | | China | | |
| JN255976 | | China | | |
| JN255975 | | China | | |
| JN255974 | | China | | |
| JN255973 | | China | | |
| JN173303 | | China | | |
| JN173302 | | China | | |
| JN173301 | | China | | |
| JN173300 | | China | | |
| JN173299 | | China | | |
| JN173298 | | China | | |
| JN173297 | | China | | |
| JN173296 | | China | | |
| JN173295 | | China | | |
| JN173294 | | China | | |

| GenBank accession no. | Strain name | Country | Genogroup | Reference |
|---|---|---|---|---|
| JN173293 | | China | | |
| JN173292 | | China | | |
| JN173291 | | China | | |
| JN173290 | | China | | |
| JN173289 | | China | | |
| JN173288 | | China | | |
| JN173287 | | China | | |
| JN173286 | | China | | |
| JN173285 | | China | | |
| JN173284 | | China | | |
| JN173283 | | China | | |
| JN173282 | | China | | |
| JN173281 | | China | | |
| JN173280 | | China | | |
| JN173279 | | China | | |
| JN173278 | | China | | |
| JN173277 | | China | | |
| JN173276 | | China | | |
| JN173275 | | China | | |
| JN173274 | | China | | |
| JN173273 | | China | | |
| JN173272 | | China | | |
| JN173271 | | China | | |
| JN173270 | | China | | |
| JF700126 | | China | | |
| JF690780 | | China | | |
| Z14976 | | | G1 | (*5*) |
| **KR270436** | **BM1** | **Korea** | | **This study** |

*Information about strain name and country were extracted from Genbank. For some entries, the corresponding information was not available. Genogroups for some strains were obtained from the cited reference. PEDV, porcine epidemic diarrhea virus.

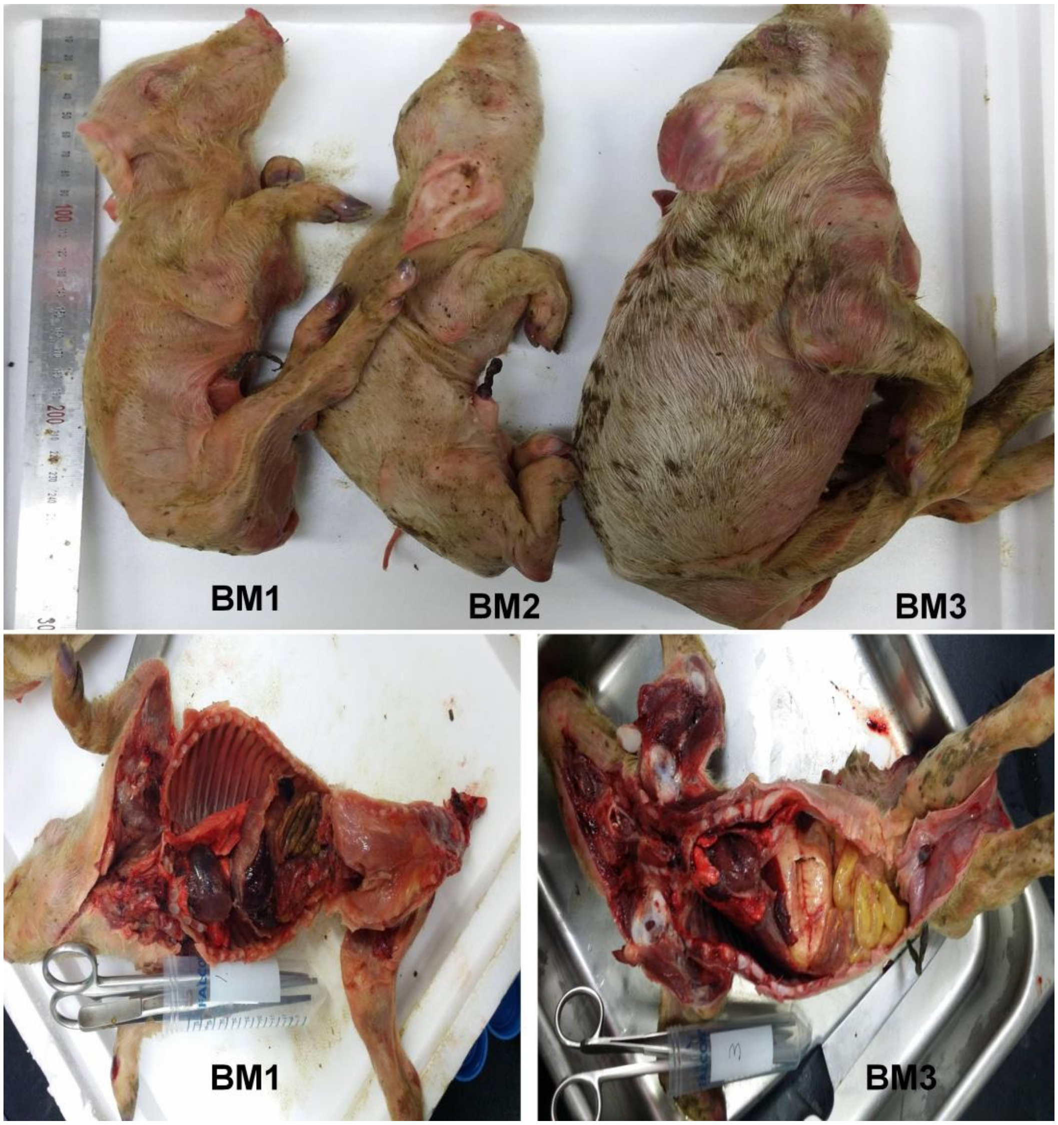


**Technical Appendix Figure 1.** Gross lesions of dead piglets that had severe diarrhea; pigs came from BM farm.

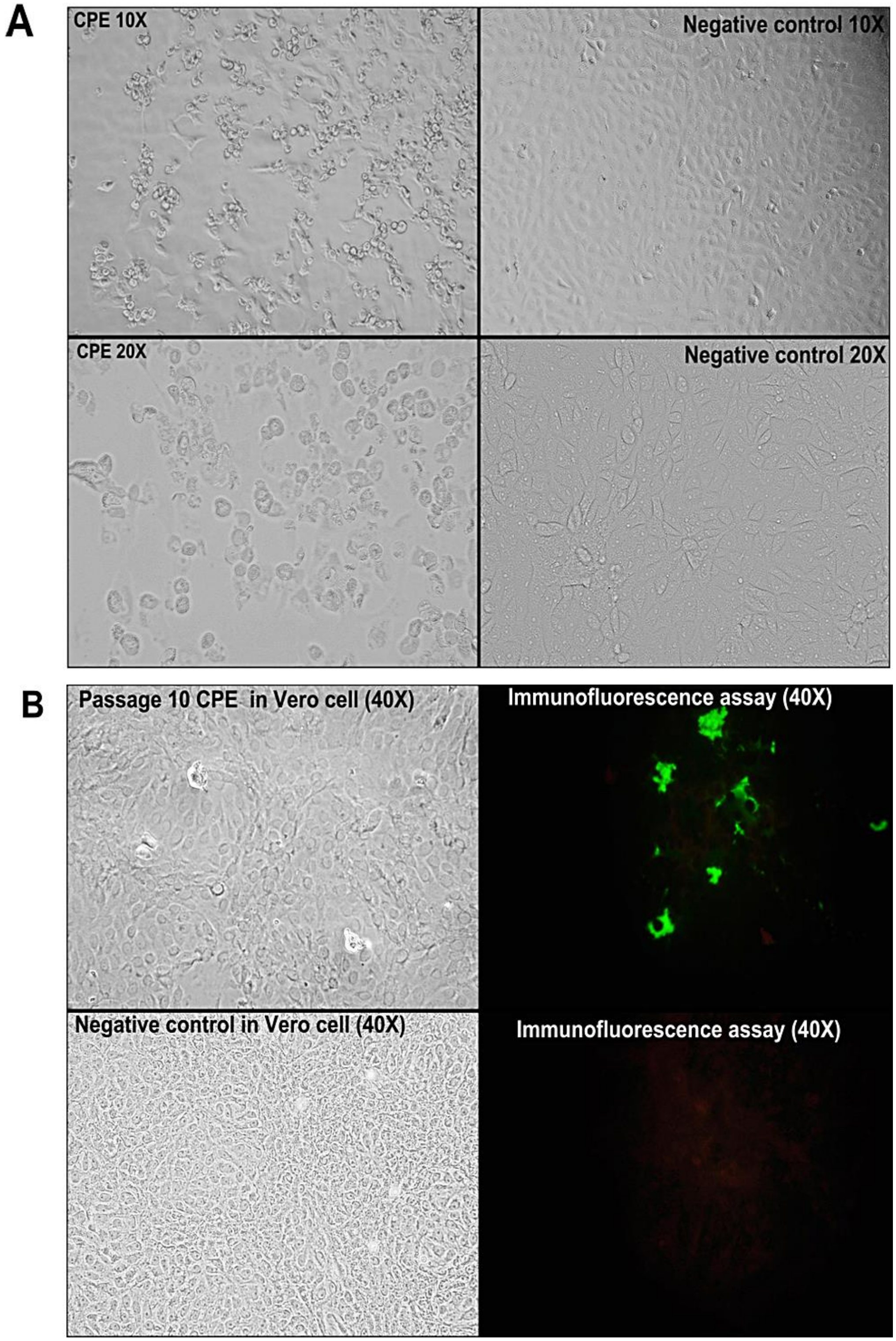


**Technical Appendix Figure 2.** Results of microscopic examination and specific IFA staining of Vero cells infected by BM1 isolate of porcine epidemic diarrhea virus at passage level 10. A) Cytopathic effects and B) Immunofluorescence assay.

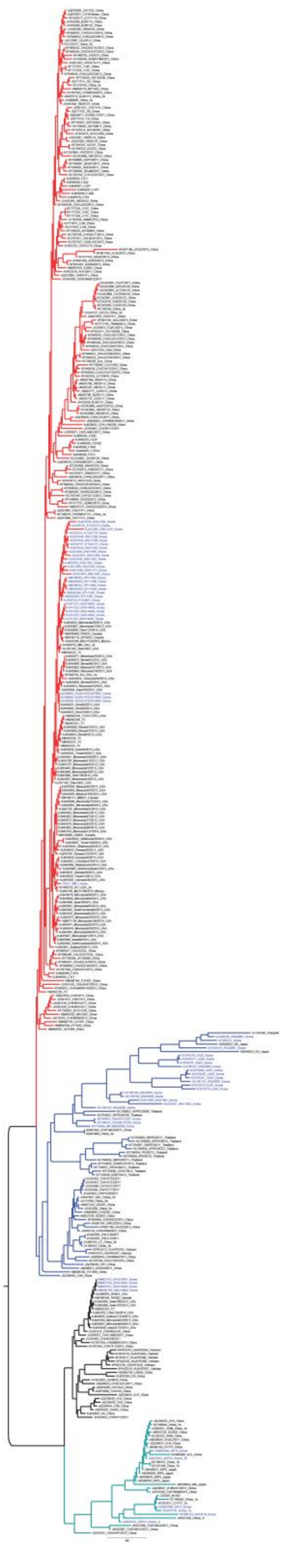

**Technical Appendix Figure 3.** Maximum likelihood phylogenetic tree of porcine epidemic diarrhea viruses, constructed on the basis of codon alignment of complete S genes.

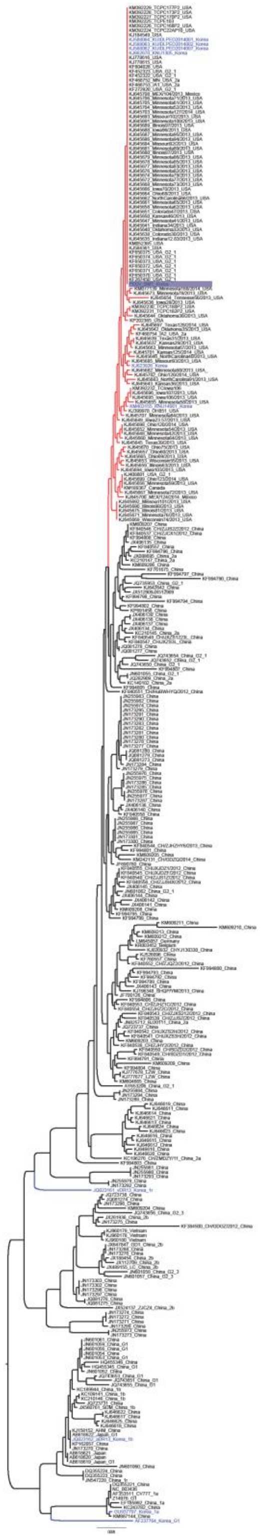

**Technical Appendix Figure 4.** Maximum likelihood phylogenetic tree of porcine epidemic diarrhea viruses, constructed on the basis of codon alignment of complete N genes.
